# Synthesis of Two Methotrexate Prodrugs for Optimizing Drug Loading into Liposomes

**DOI:** 10.3390/pharmaceutics13030332

**Published:** 2021-03-04

**Authors:** Valentina Di Francesco, Martina Di Francesco, Paolo Decuzzi, Roberto Palomba, Miguel Ferreira

**Affiliations:** 1 Laboratory of Nanotechnology for Precision Medicine, Fondazione Istituto Italiano di Tecnologia, Via Morego 30, 16163 Genoa, Italy; valentina.difrancesco@iit.it (V.D.F.); martina.difrancesco@iit.it (M.D.F.); paolo.decuzzi@iit.it (P.D.); 2 Department of Informatics, Bioengineering, Robotics and System Engineering, University of Genoa, Via Opera Pia 13, 16145 Genoa, Italy

**Keywords:** nanomedicine, drug delivery, methotrexate, prodrug, fitting and release profile, liposomes

## Abstract

Methotrexate (MTX), a compound originally used as an anticancer drug, has also found applications in a broad variety of autoimmune disorders thanks to its anti-inflammation and immunomodulatory functions. The broad application of MTX is anyway limited by its poor solubility in biological fluids, its poor bioavailability and its toxicity. In addition, encapsulating its original form in nanoformulation is very arduous due to its considerable hydrophobicity. In this work, two strategies to efficiently encapsulate MTX into liposomal particles are proposed to overcome the limitations mentioned above and to improve MTX bioavailability. MTX solubility was increased by conjugating the molecule to two different compounds: DSPE and PEG. These two compounds commonly enrich liposome formulations, and their encapsulation efficiency is very high. By using these two prodrugs (DSPE-MTX and PEG-MTX), we were able to generate liposomes comprising one or both of them and characterized their physiochemical features and their toxicity in primary macrophages. These formulations represent an initial step to the development of targeted liposomes or particles, which can be tailored for the specific application MTX is used for (cancer, autoimmune disease or others).

## 1. Introduction

Methotrexate, 2,4-diamino-N10-methyl propylglutamic acid (MTX), is a folic acid antagonist, widely used as therapeutic agent [1,2]. The molecule is a weak dicarboxylic acid with a molecular weight of 454.5 g/mol. It possesses pKa values of 4.7–5.5 and low permeability (Clog P = 0.53) with poor aqueous solubility (0.01 mg/mL). The first form of MTX, known as amethopterin, was originally synthesized in 1947. In the following years, after a slight modification of its chemical structure, it was first applied for the treatment of life-threatening neoplastic diseases (acute lymphoblastic leukemia, breast cancer and choriocarcinoma) [3,4]. Being an analog and antagonist of folic acid, MTX competes for the binding site of folate on dihydrofolate reductase (DHFR), an enzyme required in the process of DNA and RNA production [5,6,7]. At lower dosages (1:50–1:100), it also found application in a series of other diseases: Rheumatoid Arthritis (RA), Multiple Sclerosis, Vasculitis, Systemic Lupus Erythematosus, Psoriasis, Inflammatory Bowel Disease and Juvenile Idiopathic Arthritis [5,8]. It is widely accepted that the positive effects in the treatment of RA depend on MTX anti-inflammatory and immunomodulatory activity. These many applications of MTX are limited by the intrinsic features of the molecule, which impede harnessing the full potential of this drug. As mentioned before, MTX possesses poor water solubility and low permeability, which determines its low bioavailability [3,9]. Its uptake by cells is in fact extremely limited as demonstrated by in vitro assays [10]. Upon administration, MTX is rapidly excreted by the kidneys, showing a short half-life, and its plasma concentration drops rapidly upon intravenous administration, being nearly undetectable after only 4 h [2,11,12]. For the mentioned reasons, out of a discrete administered dose of drug, the amount effectively reaching its biological target tissues is supposed to be very low. Moreover, even when used at low dosages, MTX is not free from drug toxicity; rather than inefficacy, toxicity is the main cause of MTX treatment withdrawal [4]. MTX toxic effects can be severe and include hepatotoxicity, liver fibrosis, acute pneumonitis, neurotoxicity and kidney damage, just to cite some [13,14,15,16]. This considered, to optimize its use, it would be beneficial to develop novel formulation and targeted therapies to maximize its therapeutic effect, reduce its dosage and thus its toxic effects.

In recent years, various Novel Drug Delivery Systems (NDDS), such as microemulsions [17], nanoconjugates [18], nanoparticles [19], nanocapsules [20], polymeric micelles [21], pH-responsive polymersomes [22] and liposomes [10,23], have been proposed. The development of these nanoformulations allowed for the introduction of novel targeting and release strategies and the improvement of MTX loading through some modification of the molecule. Despite these valuable advancements, MTX loading still remained suboptimal, and a series of characterizations and studies are needed to approve and safely apply the majority of these novel technologies. Among the listed formulations, liposomes represent one of the most common nanocarriers for targeted and untargeted delivery [24]. They are mainly constituted by endogenous compounds, represent a very well tolerated drug delivery system, and are generally considered as pharmacologically inactive. These formulations currently represent a more efficient and less harmful alternative to conventional chemotherapy and possess the potential to positively influence therapeutic efficacy and reduce drug toxicity [25]. Their use improves the biodistribution of therapeutics to the diseased site, increases cell uptake and stabilizes the vectored compounds by protecting them from degradation and early inactivation [26]. Their biomedical application has improved the therapy of a broad variety of pathologies, and their continued translation success is progressing over time [26,27].

In this work, we modified an MTX molecule with the aim to further improve its loading into liposomes. In particular, we generated two prodrugs by covalently binding MTX to DSPE (DSPE-MTX) or polyethylene glycol (PEG-MTX). Three different liposomal formulations were obtained by loading the two prodrugs into liposomes, individually or in combination. The physio-chemical features of these MTX-Liposomal formulations, generated by loading into liposome DSPE-MTX, PEG-MTX or both were acquired and compared. The stability of the formulations was analyzed in neutral or acidic environment, and the prodrug release profile was analyzed. The uptake of Cy5 loaded liposomes in primary Bone Marrow Derived Macrophages (BMDMs) from a rat was studied: confocal imaging, revealing liposome internalization and flow cytometry analysis calculating the percent of cells positive for liposomes uptake are shown. In addition, a toxicity analysis was performed in BMDMs, in order to assess any possible differences among the formulations.

## 2. Results and Discussion

### 2.1. MTX Prodrug Synthesis and Characterizations

**Figure 1:**
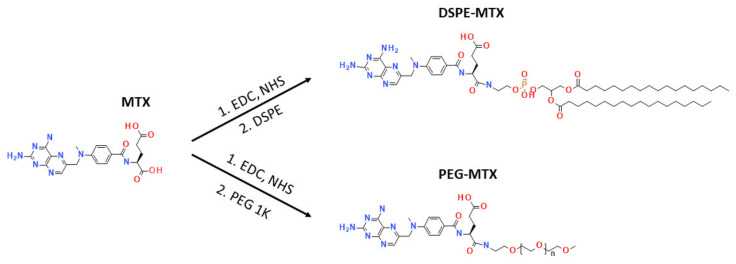
Synthesis of DSPE-MTX and PEG-MTX.

To improve MTX solubility and loading efficiency into liposomes, two different prodrugs, DSPE-MTX and PEG-MTX, were developed (Figure 1). The two molecules were chosen since they are easily incorporated into liposomes and other nanoparticles and are commonly part of the formulations themselves. The size of the two molecules was kept similar, PEG: 1 KDa and DSPE: 0.748 KDa. For the synthesis of both DSPE-MTX and PEG-MTX, MTX was pre-activated with a mixture of EDC and NHS, before the conjugation with 0.98 eq. DSPE-NH2 or PEG-1k-NH2. A low amount of the substituent was used to avoid the conjugation in MTX second carboxylic position. TLC analysis did not show any signal of the bi-substitute product. The absence of such compound is most likely due to the steric hindrance created by the bigger size of the substituent in the vicinity of the second reaction point. Product purification was achieved through precipitation with diethyl ether. Both products were obtained with a yield >75%. A scheme of the molecules and of the abovementioned reactions is available in Figure 1. Table 1 reports the formula weight, the exact mass and the molecular formula and log P values of the three compounds.

Log P is defined as the logarithm of a particular ratio of the concentrations of a solute between two solvents (for instance, for an octanol–water partition), specifically for un-ionized solutes. Three different software (Biovia, VCCLab and ACD ChemSchetch) were used to analyze this parameter for MTX and the two generated prodrugs. The software used a different combination of algorithms to perform the calculation. Biovia is based on an algorithm which considers the ionization states of the molecule [28]; log P is calculated using pKa information for each atom in the molecule. VCCLab (ALOGPs) was developed with 12908 molecules from the PHYSPROP database using 75 E-state indices. In total, 64 neural networks were trained using 50% of molecules selected by chance from the whole set. The logP prediction accuracy is the root mean squared error, rms = 0.35, and standard mean error s = 0.26 [29]. ACDLabs uses a consensus model for the determination of log P. Applying both classic algorithms (based on >12,000 experimental log P values, by using the principle of isolating carbons) and GALAS algorithms (based on a training set of >11,000 compounds, which provides a value for log P that is adjusted with data from the most similar compounds), the consensus algorithm weights the calculation to the model best suited for each structure [30]. Results are presented in Table 1 and reported here for convenience, respecting the software order indicated above. The following results were obtained for MTX: log P. = 0.11, −0.91, 0.023; for DSPE-MTX: log P. = 13.84, 7.53, 16.63; and for PEG-MTX: log P. = −0.67; 0.42; −0.43. The similar results obtained for MTX and PEG-MTX indicate that these molecules can be equally dissolved in water and organic solvents. While for MTX-DSPE, regardless of the discrepancy between VCCLab software results and the two other software (to be ascribed to the different combination of algorithms used), all three programs show that MTX-DSPE is mainly soluble in organic solvents. The higher value of log P for MTX-DSPE is due to the two aliphatic chains of the lipid. These aliphatic chains are very hydrophobic and led the software to predict MTX-DSPE to be mainly soluble in the organic phase. Instead, the amphiphilic behavior of PEG chain led the software to predict that the chain will not influence MTX solubility.

Experimentally, MTX has shown low solubility in water and organic solvents, such as dichloromethane and chloroform, which are commonly used to prepare liposomes. The theoretical MTX-PEG log P (log P. = −0.67, 0.42, −0.43) could indicate similar solubility features for this compound and naïve MTX (log P. = 0.11, −0.91, 0.023). Nonetheless, MTX-PEG showed a more hybrid behavior regarding these solvents, revealing to have higher solubility in water and organic solvents with respect to MTX. This is due to the amphiphilic properties of this prodrug, which can spontaneously organize in small structures, accordingly to the solvent used. This factor is probably not taken into consideration by the software algorithms. Similar amphiphilic behavior is observed for MTX-DSPE (log P. = 13.84, 7.53, 16.63) for the same capability of self-organizing into small structures; it was possible to dissolve DSPE-MTX at a low concentration in water, despite the log P. results. The observed behaviors allowed the loading of both prodrugs into the liposomes with high yielding; conversely, MTX direct loading was not successful.

### 2.2. MTX Liposome Assembly and Characterization

**Figure 2:**
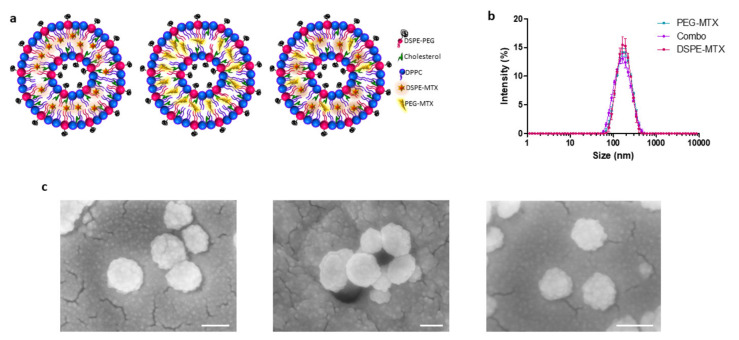
(**a**) Schematic representation of liposomes. (**b**) Hydrodynamic diameter of PEG-MTX-LIP, DSPE-MTX-LIP and Combo-LIP via dynamic light scattering analysis. (**c**) Scanning electron microscopy images of PEG-MTX-LIP, DSPE-MTX-LIP and Combo-LIP, respectively (scale bar: 100 nm).

Liposomes were synthesized via the thin layer evaporation method (TLE), using DPPC; cholesterol; carboxyl-terminated DSPE-PEG chains; and the two prodrugs: DSPE-MTX and PEG-MTX (Figure 2). The DSPE-MTX and PEG-MTX were added during the lipid film formation phase. It might be speculated that the DSPE-MTX could intercalate with the DPPC and DSPE-PEG chains, considering that the lipid part of DSPE-MTX is identical to DSPE-PEG and similar to DPPC [31]. Regarding the localization of PEG-MTX, there are two options: it could be intercalated into the lipid membranes, with a similar configuration reported for DSPE-MTX, or in the inside of the phospholipid bilayer. The loading of the combination of both prodrugs increases the complexity of the allocation, making even harder to produce hypothesis on their possible disposition inside the liposome. A representative schematic of the putative structures of the three liposomes is proposed in Figure 2a.

DLS analysis showed average hydrodynamic diameters of 159 ± 3.0 nm, 166 ± 0.6 nm and 148 ± 1.0 nm for the DSPE-MTX-LIP, PEG-MTX-LIP and Combo-LIP, respectively (Figure 2b and Table 2). A similar size was reported for empty liposomes (157.8 ± 2.2 nm), confirming that lipophilic drug encapsulation cannot affect particle size [32]. All the formulations are characterized by a very homogenous population: with a polydispersity index (PDI) of 0.15 (Table 2). Images of the formulations were acquired by SEM (Figure 2c) and TEM analyses (Figure S3), confirming formulations sphericity and size. Liposomes presented a negative surface electrostatic ζ-potential of −38 ± 0.26 mV, −41 ± 0.4 mV and −41.3 ± 3 mV for the DSPE-MTX-LIP, PEG-MTX-LIP and Combo-LIP, respectively (Table 2). It is important to note that the unchanged surface electrostatic ζ-potential found for PEG-MTX-LIP supports the hypothesis of the localization of the compound between the two membranes.

To evaluate the encapsulation efficiency (EE) of MTX inside liposomes, high-performance liquid chromatography (HPLC) was used. The EE was calculated as the percentage ratio between the drug loaded mass and the drug input mass, used during nanoparticle synthesis. For DSPE-MTX-LIP, the encapsulation efficiency was equal to 79.9 ± 5.6% (799 ± 56 µg), while for PEG-MTX-LIP: 82 ± 7.5% (820 ± 75 µg) and the Combo-LIP: 80.2 ± 1.8% (802 ± 18 µg) as reported in the Table 2. The direct loading of MTX unmodified molecule into liposomes was extremely difficult due to the extremely poor solubility of the compound both in water and organic solvents as also reported elsewhere [31,33]. A series of MTX modification-based strategies have been pursued by other groups in recent years. For example, Guimarães et al. produced an MTX sodium salt and loaded it into liposomes through the ethanol injection method achieving an EE% equal to 32% [23]. In another study, Li et al. synthetized an MTX prodrug by conjugating the drug to a phospholipid (PC) achieving an EE% equal to 20.7 ± 2.4% [10]. Our results reveal that the approach proposed in this work, based on the use DSPE-MTX and PEG-MTX, led to achieving a significantly higher EE (around 80%). Such a result represents a considerable step forward in the encapsulation of MTX into liposomes.

**Figure 3:**
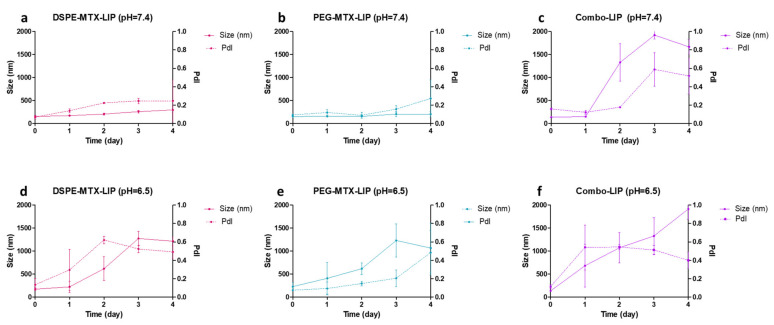
(**a**–**c**) Stability of all the formulations at pH = 7.4 and (**d**–**f**) at pH = 6.5.

In order to investigate liposome stability under the conditions found in vivo (37 °C), two buffer solutions were used to reproduce the in vitro physiological condition (pH 7.4) and mildly acidic microenvironment (pH 6.5) typical of malignant solid tumors [34], and of inflamed tissues [35,36]. Liposome size and size distribution were monitored over a period of 4 days. As reported in Figure 3a–c, both DSPE-MTX-LIP and the PEG-MTX-LIP showed to be stable at 37 ± 2 °C and pH = 7.4 with a percentage change in size and PDI lower than 15% for the entire observation period. The Combo-LIP showed an increase in size and PDI already after the first day, indicating a formulation instability, which was also evident for the entire observation period. A different behavior was documented at pH = 6.5 (Figure 3d–f). All three liposomal formulations resulted to be unstable under a slightly acidic environment, with a rapid size increase. The DSPE-MTX formulation resulted to be more stable than the other two after one day of observation. In any case, it is possible to conclude that a slight decrease in pH is able to destabilize the three formulations, possibly also leading to a faster release of the prodrugs from the liposomes. This last consideration might be particularly relevant with the vision of using these vectors for the therapy of cancer and other inflammatory diseases MTX is used for. By exploiting the slight acidic environment, characterizing malignant tumors and inflamed areas, drug release could be fostered in those areas rather than in healthy tissues.

In order to investigate liposomes stability under the conditions found in vivo (37 °C); two buffer solutions were used to reproduce an in vitro physiological condition (pH 7.4) and mildly acidic microenvironment (pH 6.5) typical of malignant solid tumors [34], and of inflamed tissues [35,36]. Liposome size and size distribution were monitored over a period of 4 days. As reported in Figure 3a–c, both DSPE-MTX-LIP and the PEG-MTX-LIP showed to be stable at 37 ± 2 °C and pH = 7.4 with a percentage change in size and PDI lower than 15% for the entire observation period. The Combo-LIP showed an increase in size and PDI already after the first day, indicating a formulation instability, which was also evident for the entire observation period. A different behavior was documented at pH = 6.5 (Figure 3d–f). All the three liposomal formulations resulted to be unstable under a slightly acidic environment, with a rapid size increase. DSPE-MTX formulation resulted to be more stable than the other two after one day of observation. In any case, it is possible to conclude that a slight decrease in pH is able to destabilize the three formulations, possibly also leading to a faster release of the prodrugs from the liposomes. This last consideration might be particularly relevant with the vision of using these vectors for the therapy of cancer and other inflammatory diseases MTX is used for. By exploiting the slight acidic environment, characterizing malignant tumors and inflamed areas, drug release could be fostered in those areas rather than in healthy tissues.

### 2.3. MTX-Liposome Release Profiles

**Figure 4:**
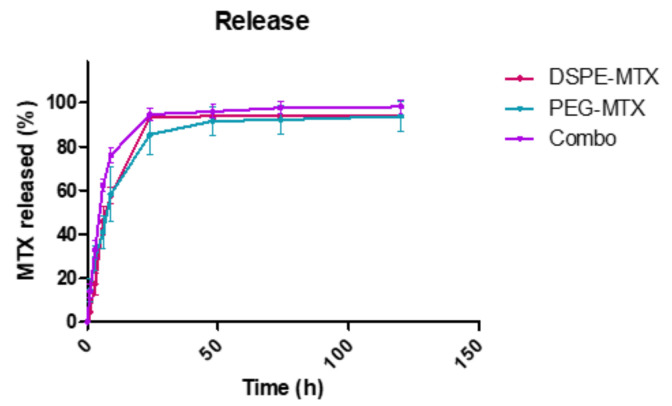
In vitro release profile of DSPE-MTX (red line), PEG-MTX (light green line) and their combinations (purple line) Figure 3. Different experiments ± standard deviation (SD).

The release profiles of DSPE-MTX, PEG-MTX and Combo liposomes were determined under infinite sink conditions (4 L release volume). Briefly, the three liposomal formulations were placed in 4 L of PBS buffer (1×, pH 7.4) at 37 ± 2 °C under magnetic stirring. Three samples for each time point were collected, destroyed with cold methanol and left to dry. The obtained powder was dissolved in AcN/H_2_O (1:1, *v*/*v*) to release the remaining DSPE-MTX and PEG-MTX for HPLC analysis. The three liposomal formulations showed similar biphasic kinetics with different percentages of drug released (Figure 4). Specifically, DSPE-MTX and PEG-MTX formulations showed a burst drug release within the first 9 h, with approximately 60% rapid release. The Combo formulation exhibited a faster release with 75% of DSPE-MTX/PEG-MTX after 9 h. The faster release was also supported by stability data relative to this formulation (Figure 3c). The Combo liposome size and polydispersity index increased after 1 day, confirming the lower stability of this formulation. The remaining portion of drugs was slowly and continuously released over time, yielding a ~95% release after 1 day for Combo and 3 days for both DPSE-MTX or PEG-MTX. The initial phase release under sink conditions is likely associated with drug molecules closer to the particle surface. These molecules diffuse out more rapidly and over a short distance upon exposure to a release medium in vitro or extracellular fluid in vivo. To better understand which kinetic model better described the three different liposomal formulation release profiles, experimental data were fitted on various mathematical models: zero order, first order, Higuchi, Korsemeyer–Peppas and Weibull [37,38]. The cumulative % drug released versus time, the log cumulative % drug remained versus time, the cumulative % drug released versus the square root of time plot and the log cumulative % drug released versus the log time plot [38] for all the three formulations are reported in Figures S4–S6. Their correlation coefficient (R2) values are reported in the Table 3. According to data, all the models provided an accurate fitting for the three release profiles, with some differences. All three formulations followed a Korsemeyer–Peppas law (highest correlation coefficient R^2^ value): DSPE-MTX showed an MTX non-Fickian diffusion (super case-II transport mechanism) (n ≥ 0.85) [39], while PEG-MTX and DPSE-MTX/PEG-MTX a non-Fickian diffusion (anomalous transport) (0.43 ≤ n ≤ 0.85) [39]. On the contrary, the b value obtained (b ≥ 0.75) with the Weibull equation suggested a super case-II transport mechanism for all three formulations. Furthermore, obtained data suggested that multiple mechanisms, such as diffusion and erosion [40], act simultaneously during the release study for all the three liposomal formulations. This could depend on the different interactions of prodrugs with lipids and PEG-lipids in the liposome structure previously reported for guanosine [41]. Similar non-Fickian diffusion was reported for other drugs delivered using liposomes [42,43,44,45,46].

### 2.4. Liposome Uptake

**Figure 5:**
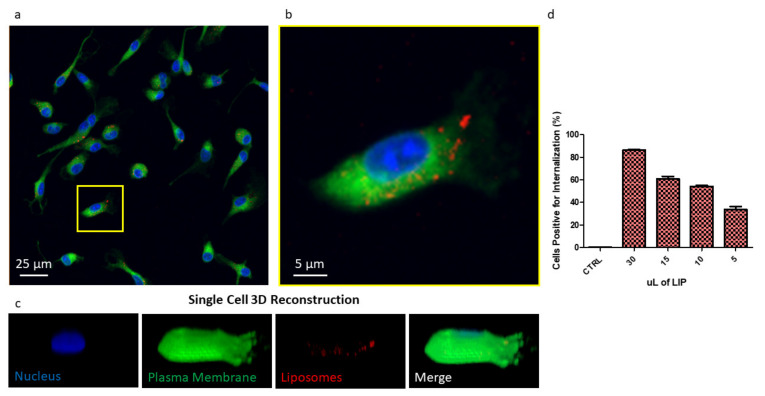
(**a**) Image reporting a maximum intensity profile of a z-stack of BMDMs treated with liposome reporting in blue the nuclei, in green the plasma membrane and in red the liposomes. (**b**) Higher magnification inset of a single cell. (**c**) Single cell 3D reconstruction with split channels and merge. (**d**) Flow cytometry analysis of BMDM uptaking Cy5-liposomes.

In order to investigate liposome cell uptake, liposomes were loaded with the tracer molecule DSPE-Cy5. The resulting liposomes (Cy5-LIP) were found to be comparable in size and ζ-potential with the other formulation presented in this paper, as shown in Figure S7A. Their release profile revealed DSPE-Cy5 is slowly released over time (Figure S7B), indicating that this formulation possesses suitable characteristics for imaging purposes. BMDMs were treated with Cy5-LIP. Confocal imaging showing Cy5-LIP (red signal) internalized into BMDMs is reported in Figure 5a as the maximum intensity profile of a z-stack. From the figure, liposomes appeared to be within the plasma membrane (green signal); the nucleus was stained by DAPI (blue signal). An image reporting each of the acquired channels is presented in Figure S8. A high magnification image of one cell was also acquired (Figure 5B). From this image, it is possible to clearly appreciate that Cy5-LIP were relatively uniformly disposed inside the cell cytosol. A single cell 3D reconstruction is shown in Figure 5c. The image reports a reconstruction of the surface for each of the acquired channels. The surface reconstruction confirms Cy5-LIP internalization: in the merged image, a minor Cy5 signal was retrieved on the BMDM membrane, indicating that most of the liposomes were found inside the cell, while only a minor portion is on the cell surface, possibly while being uptaken.

Flow cytometry analysis revealed that the uptake of Cy5-LIP is dose-dependent (Figure 5d). By increasing the amount of liposomes used for BMDM treatment, the percent of cells positive for internalization also increases. Treating BMDMs with 5 µL of Cy5-LIP suspension, 33% of cells were found to be positive for internalization; the percentage increased to 54.2% and 60.7% when BMDMs were treated with 10 µL and 15 µL, reaching its maximum (86.4%) when 30 µL of liposome suspension was used. All the treatments were performed for 30 min.

Taken together, these data indicate that liposomes were easily uptaken by BMDMs and that at the considered time point, 33% of cells are already positive for internalization when using a small amount of liposomes. Only very few liposomes were found on the cell membrane, probably taken in the process of being internalized. These observations confirm that this kind of formulation can easily penetrate the cell membrane to deliver its payload (in this case, represented by Cy5, which was used as a tracer). Considering the very low solubility of MTX in its naïve forms, using a liposomal formulation is thus expected to favor its cell penetration. MTX dispersion inside body fluids, its circulation half-life (as also reported elsewhere [10,47]) and thus its availability to the biological target [12] are supposed to be improved. Moreover, it is important to underline that liposomes can be easily functionalized with targeting ligands, imaging agents, small molecules, peptides, proteins, antibodies [26] and also aptamers [48]. This versatility should also allow to tailor our MTX liposomes based on the specific pathology to treat.

### 2.5. Cell Cytotoxicity Analyses

**Figure 6:**
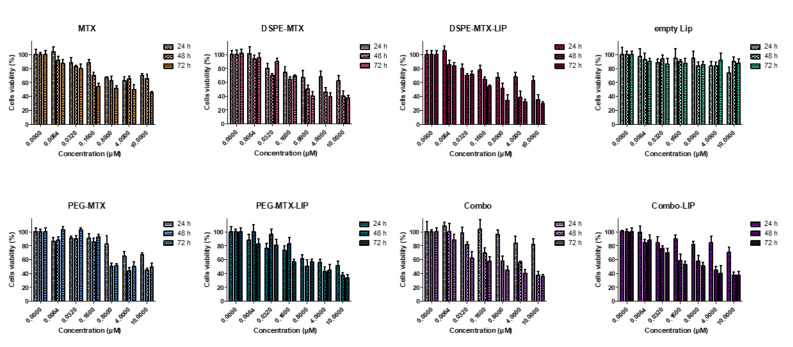
Viability of BMDMs incubated with MTX, DSPE-MTX, DSPE-MTX-LIP, empty LIP, PEG-MTX, PEG-MTX-LIP, Combo and Combo-LIP at 3 different time points.

To evaluate the cytotoxicity of the prodrugs and prodrug-loaded-LIP, cell viability was measured using the MTT assay. This assay calculates the reduction of MTT by mitochondrial dehydrogenase to blue formazan product, which reflects the normal function of mitochondria. Hence, the measurement of cytotoxicity and cell viability was obtained. Different concentrations of free MTX, DSPE-MTX and/or PEG-MTX, DSPE-MTX-LIP, PEG-MTX-LIP and Combo-LIP with a drug concentration ranging from 60 nM to 10 µM were tested on BMDMs for different time points, 24, 48 and 72 h. Empty liposomes were tested as a control. For the treatment with free MTX, it is important to note that the molecule was dissolved in DMSO, considering the difficulty of dissolving it in culturing media. Viability plots are presented in Figure 6. IC_50_ values calculated at 72 h are reported in Table 4. MTX IC_50_ was found to be equal to 2.41 ± 0.14 µM. DSPE-MTX, free or loaded into liposomes, was found to have a slightly higher toxicity (IC_50_ = 0.9 ± 0.1 µM and 0.7 ± 0.12 µM, respectively). A minor difference in IC_50_ was found for free or loaded PEG-MTX (IC_50_ = 2.5 ± 0.08 µM and 1.6 ± 0.1 µM, respectively). The coadministration of the two prodrugs and the administration of combo liposomes showed an IC_50_ very similar to DSPE-MTX: IC_50_ values for the combo were found to be equal to 0.6 ± 0.1 µM and 0.9 ± 0.1 µM (free prodrugs and combo-LIP, respectively). These results were in agreement with results obtained in other works produced by our group and others [31,45,49]. In summary, a slight difference in IC_50_ among MTX, DSPE-MTX, PEG-MTX and the liposomal formulation derived was found. This finding supports the hypothesis that the activity of the molecule is possibly maintained, despite the changes operated in the structure.

## 3. Conclusions

In the present manuscript, a strategy to efficiently load a high content of MTX into liposomes is presented. Two different prodrugs were generated by binding the compound to DSPE and PEG. The modifications operated to the molecule positively influenced MTX solubility. While the loading of the naïve molecule is particularly inefficient, the two prodrugs can be easily and directly loaded into the liposomes, singularly and in combination. The generated formulations turned out to be comparable in terms of physiochemical features, presenting a similar size of ~155 nm, a narrow size distribution and a mean surface charge of about −40 mV. At physiologic pH, DSPE-MTX and PEG-MTX liposomes were found to be more stable than the formulation comprising both the prodrugs. At slightly acidic pH, all the formulations showed to be unstable after one day of observation. As for the release, all three liposomal formulations showed a biphasic release; both mechanisms of diffusion and erosion are involved in the process, as demonstrated by the mathematical fitting. The data acquired at confocal and by flow cytometry confirmed the suitability of liposomes in granting cell uptake. Considering that DSPE-MTX and PEG-MTX are constituents of the liposomes structure, a higher MTX uptake is expected if compared to the naïve molecule. These considerations, taken together with the benefits offered by liposomal formulations (i.e., extended circulation half-life and favored accumulation at the diseased site), support the potential advantages of MTX liposomes as a safe and efficient drug delivery system for a multitude of diseases in which MTX is successfully used.

## 4. Materials and Methods

### 4.1. Materials

1-Ethyl-3-(3-dimethylaminopropyl) carbodiimide (EDC), *N*-Hydroxysuccinimide (NHS) and Triethylamine (TEA) were purchased from Sigma-Aldrich (St. Louis, MO, USA). Methotrexate (MTX) and NH2-PEG (1K) were bought by AlfaAesar (Haverhill, MA, USA). 1,2-distearoyl-sn-glycero-3-phosphoethanolamine-*N*-[succinyl(polyethylene glycol)-2000] (DSPE-PEG-COOH), 1,2-distearoyl-sn-glycero-3-phosphoethanolamine (DSPE-NH2) and 1,2-Dipalmitoyl-sn-glycero-3-phosphocholine (DPPC) were purchased from Avanti Polar Lipid (Alabaster, AL, USA). All reagents and solvents were used without further purification. Cy5 was purchased from Luminoprobe (Hunt Valley, MD, USA).

### 4.2. Synthesis of DSPE-MTX

DSPE-MTX was synthesized as reported by Ferreira and coworkers with some modifications [49]. Briefly, 30 mg of MTX was incubated with 1 eq. of 1-Ethyl-3-(3-dimethylaminopropyl)carbodiimide (EDC) and 1 eq. of *N*-Hydroxysuccinimide (NHS) in 2 mL Dimethyl sulfoxide (DMSO) for 30 min, at room temperature. An amount of 0.98 eq. DSPE-NH2 dissolved in 0.5 mL DMSO was added. The reaction was left to stir for 72 h after adding a catalytic amount of triethylamine (TEA). The mixture was washed three times with cold diethyl ether. Finally, the prodrug was lyophilized and stored at −20 °C.

### 4.3. Synthesis of PEG-MTX

For PEG-MTX synthesis, 20 mg of NH2-PEG (1000 Da) was dissolved in a mixture of dichloromethane (DCM) and MeOH (2:1 Ratio). An amount of 0.98 eq. of MTX was dissolved in 200 mL of dimethylformamide (DMF) and added to the previous solution. A catalytic amount of triethylamine (TEA) was added to the reaction and was left to stir for 16 h. The intended product was precipitated with cold diethyl ether, and then washed 3 times with cold diethyl ether to obtain the final product with a yield of 90%. More details of this procedure can be found in the previous literature [49].

### 4.4. Synthesis of DSPE-Cy5

DSPE-Cy5 was synthetized as reported elsewhere [49]. Briefly, DSPE-NH2 (15 mg) was dissolved in dichloromethane (DCM)/cethanol (MeOH), 2:1 *v*/*v*. Cyanine-5 NHS ester (0.98 eq.) was dissolved in 200 mL of dimethylformamide (DMF) and added to the previous solution. Triethylamine (TEA) was added in order to catalyze the reaction; stirring was maintained for 16 h. Cold diethyl ether was used for precipitating the product, which was then washed three times with cold diethyl ether obtaining the final compound with a yield equal to 90%.

### 4.5. Determination of Log P

DSPE-MTX and PEG-MTX Log P were determined with the help of three computational softwares: Biovia Draw, DASSAULT SYSTEMES, https://www.3ds.com/ (accessed on 5 January 2021); VCCLAB, Virtual Computational Chemistry Laboratory, http://www.vcclab.org, (accessed on 5 January 2021) 2005; and ChemSketch, ACD/LABS; https://www.acdlabs.com/ (accessed on 5 January 2021). Log P. is given by the following equation:

(1) $$\log P_{\mathrm{oct}/\mathrm{wat}}\;=\log\left(\frac{{\left[\mathrm{solute}\right]}_{\mathrm{octanol}}}{{\left[\mathrm{solute}\right]}_{\mathrm{water}}}\right)$$

### 4.6. Synthesis of MTX Liposomes (MTX-LIP) and Cy5 Liposomes (Cy5-LIP)

Liposomes (LIP) were prepared by thin-layer evaporation (TLE) [31]. Briefly, DPPC, cholesterol, DSPE-PEG (6:3:1) (total amount: 40 mg) and DSPE-MTX/PEG-MTX or both prodrugs (1 mg of prodrug) were dissolved in chloroform in a round-bottomed flask. After the evaporation of the organic solvent at 60° under reduced pressure, the lipid film was left under the hood overnight to remove any traces of residual solvent. The lipid film was hydrated with 2 mL of HEPES buffer (pH = 7.4, 10 mM) and then subjected to three alternate cycles (3 min each) of warming at 60 °C (thermostatic water bath) and vortexing at 700 rpm. The sample was dialyzed against HEPES buffer (pH = 7.4, 10 mM) for 1h at 4 °C. 

For the preparation of Cy5-LIP, DSPE-Cy5 was used instead of the prodrugs. Specifically, 0.3 mg of DSPE-CY5 was dissolved in chloroform together with lipids and cholesterol in a round-bottomed flask; the same procedure was followed. The purification step to remove the excess of Cy-5 was conducted by ultracentrifugation (1 h, 45,000 rpm). All formulations obtained were freshly used or stored at 4 °C overnight as concentrated dispersions.

### 4.7. Liposome Morphological Characterization

SEM characterization: Liposomes were fixed for 2 h in 2% glutaraldehyde in 0.1 M cacodylate buffer. After fixation, the samples were washed twice with the same buffer and postfixed for 1 h in 1% osmium tetroxide, in 0.1 M cacodylate buffer. After several washes with distilled water, samples were subsequently dehydrated in a graded ethanol series, 1:1 ethanol:hexamethyldisilazane (HMDS), and 100% HMDS and dried overnight. Samples were sputtered using gold. SEM images were collected using JEOL JSM-7500FA (Jeol, Tokyo, JAPAN), operating at 5 kV of accelerating voltage.

TEM Characterization: Transmission electron microscopy (TEM) micrographs were acquired using a JEOL JEM 1011 (Jeol, Japan) electron microscope operating with an acceleration voltage of 100 kV and recorded with a 11 MegaPixel fiber optical charge-coupled device (CCD) camera (Gatan Orius SC-1000). LIP was diluted 1:100, dropped on 150-mesh glow discharged “Ultrathin” carbon-coated Copper TEM grids and dried. Dried TEM samples were negatively stained using 2% uranyl acetate aqueous solution.

### 4.8. Particles Size, Surface Charge and Stability Characterizations

Particle size, size distribution and ζ-Potential of all the formulations were measured using dynamic light scattering (DLS). For stability studies, 1 mL of each formulations was put in 9 mL of PBS (pH = 7.4, 1×) or slightly acidic buffer (pH = 6.5, 1×) (final volume = 10 mL) at physiologic temperature (37 ± 2 °C), under agitation. At specific time intervals of 1, 2, 3 and 4 days, samples were taken, and their physical features were examined. 

### 4.9. Drug Loading and Release Analysis

To measure the MTX encapsulation efficiency (EE), samples were destroyed with cold methanol, left to dry, dissolved in acetonitrile (AcN)/H_2_O (1:1, *v*/*v*) and analyzed by high-performance liquid chromatography (HPLC) (Agilent 1260 Infinity, Waldbronn, Germany) equipped with a 100 μL sample loop injector. A C18 column (2.1 × 250 mm, 5 μm particle size, Agilent, Santa Clara, CA, USA) was used for the chromatographic separation. MTX was eluted under isocratic conditions using a binary solvent system [H_2_O + 0.1% (*v*/*v*) TFA and AcN + 0.1% (*v*/*v*) TFA 43:57 *v*/*v*] pumped at a flow rate of 1.0 mL/min. The ultraviolet (UV) detection was set at 430 nm.EE was determined using the following equation:

(2) $$\mathrm{EE}\;\left(\%\right)=\frac{\mathrm{MTX}\;\mathrm{weight}\;\mathrm{in}\;\mathrm{particles}}{\mathrm{MTX}\;\mathrm{inizial}\;\mathrm{feeding}\;\mathrm{amount}}\times 100$$

To study MTX and Cy5-release kinetics, 200 μL of MTX-LIP or Cy5- LIP solution was placed into Slide-A-Lyzer MINI dialysis microtubes with a molecular cutoff of 10 kDa (Thermo Scientific, Rockford, IL, USA) and dialyzed against 4 L of PBS buffer (pH 7.4, 1×). For each time point, three samples were collected and dried. LIP samples were destroyed with cold methanol, left to dry, dissolved in AcN/H_2_O (1:1, *v*/*v*) and analyzed by HPLC for the MTX. The experimental data were fitted using different mathematical models: the zero-order, the first-order, the Higuchi, the square root, the two-phase Weibull and Korsmeyer–Peppas models [37,38].

### 4.10. Cy5 Loading and Release Analysis

For determining DSPE-CY5 encapsulation efficiency (EE), liposomes were destroyed by adding cold methanol. After solvent evaporation, the destroyed formulation was dissolved in acetonitrile (AcN) and analyzed by the spectrophotometer (ʎ = 640 nm). EE was determined using the following equation:

(3) $$\mathrm{EE}\;\left(\%\right)=\frac{\mathrm{DSPE}-\mathrm{CY}5\;\mathrm{weight}\;\mathrm{in}\;\mathrm{particles}}{\mathrm{DSPE}-\mathrm{CY}5\;\mathrm{initial}\;\mathrm{feeding}\;\mathrm{amount}}\times 100$$

For studying DSPE-Cy5-release kinetic, 200 μL DSPE-Cy5-LIP suspension was placed into Slide-A-Lyzer MINI dialysis microtubes with a molecular cutoff of 10 kDa (Thermo Scientific) and dialyzed against 4 L of PBS buffer (pH 7.4, 1×). For each time point, three samples were collected and dried. Samples were subsequently destroyed with cold methanol, and solvent was left to dry. The samples were then dissolved in AcN and analyzed at the spectrophotometer following the indications used for EE determination.

### 4.11. Bone Marrow Derived Macrophages Harvesting

BMDMs from rats were cultured at 37 °C in 5% CO_2_, in high-glucose DMEM, supplemented with 15% FBS and 1% L-glutamine, according to ATCC instructions. Cells were isolated by the following procedures, also indicated elsewhere [50,51]. After sacrificing the animal, femurs were explanted, cleaned from surrounding tissues and washed in PBS (Thermo Fisher Scientific, Waltam, MA, USA), and a cut was performed at both ends. PBS was used for flushing the cavities to harvest cells, and the sample was centrifuged for 10 min at 800 RPM at 4 °C. Cells were plated in media supplemented with macrophage colony-stimulating factor (mCSF) (10 ng mL^−1^) (Sigma-Aldrich). Three days after, media were completely replaced after one wash in PBS, and the following day, cells were scraped, counted and seeded for further processing. The procedures were conducted following the guidelines of the Institutional Animal Care and Use Committee of IIT.

### 4.12. Confocal Fluorescent Microscopy Imaging

Confocal images were obtained using a Nikon-A1 confocal microscope (Nikon Corporation, Tokyo, Japan). Cy5-DSPE was used in the fabrication step of liposomes, allowing their visualization by a confocal microscope. Liposomes were suspended in HEPES buffer. 65,000 BMDMs were seeded into each well of a Nunc Lab-Tek II chamber slide system (Thermo Fisher Scientific), maintaining culturing conditions, as described above. Cells were treated with 10 µL of Cy5-LIP for 30 min. To favor the homogeneous distribution of the particles in the wells, all the treatments were performed by suspending liposomes in an adequate volume of culturing media prior of the treatment; media without liposomes were replaced by media with liposomes. After 30 min, the culturing medium was removed, and cells were washed in PBS (Thermo Fisher Scientific). Fixation was performed using a 3.7% solution of paraformaldehyde (Sigma-Aldrich) for 5 min. Cell Mask was used to stain the plasma membrane, and nuclei were stained using DAPI (Thermo Fisher Scientific). A z-stack section was acquired using a 60× objective (12 steps of 1000 nm each were acquired). The maximum intensity profile is presented in Figure 5a. Surface reconstruction of macrophages is shown in Figure 5c.

### 4.13. Cell Internalization Studies

Flow cytometry was performed using a FACS ARIA (Becton Dickinson, Franklin Lakes, NJ, USA). In total, 200,000 BMDMs were seeded into each well of a 12-well plate, maintaining culturing conditions indicated in the cell culturing section. Cells were treated for 30 min with different volumes (5, 10, 15, 30 µL) of Cy5-LIP. After treatment, cells were washed using cold PBS in order to ease the scraping procedures. Cold DMEM, high glucose, no glutamine and no phenol red (Thermo Fisher Scientific) were added, and cells were harvested by gently scraping the plastic bottom (a volume of 200 μL of was used). Samples were immediately stored in ice and vortexed right before the analysis.

### 4.14. Toxicity Analysis

BMDMs were cultured according to the conditions indicated above. Cell viability was determined using MTT assay; this assay detects the reduction of MTT [3-(4,5-dimethylthiazolyl)-2,5-diphenyltetrazolium bromide] (Sigma-Aldrich) by mitochondrial dehydrogenase to blue formazan product. Cells were seeded into 96-well plates at a density of 20 × 103 cells per well and incubated for 24, 48 and 72 h. Cells were treated with different concentrations of free MTX, DSPE-MTX, PEG-MTX, DSPE-MTX/PEG-MTX, DSPE-MTX-LIP, PEG-MTX-LIP and PEG-MTX/DSPE-MTX-LIP (namely, 0.0064, 0.032, 0.16, 0.8, 4, 10 and 0 μM of MTX), or empty LIP. For the free MTX condition, MTX was pre-dissolved in DMSO due to the impossibility to dissolve the compound in culturing media. The MTT solution was added for 4 h, and the formed formazan crystals were dissolved in ethanol. Absorbance was measured at 570 nm, using 650 nm as the reference wavelength (Tecan, Männedorf, Swiss). The percentage of cell viability was assessed according to the following equation:

(4) $$\mathrm{Cell}\;\mathrm{viability}\;\left(\%\right)=\frac{{\mathrm{Abs}}_{T}}{{\mathrm{Abs}}_{C}\;}\times 100$$

where AbsT is the absorbance of treated cells and AbsC is the absorbance of untreated cells (control).

## Figures and Tables

**Table 1:** Formula weight, exact mass, molecular formula, log P value (Bovia, VCClab, ACD chemsketh) of MTX and its prodrugs.

Compound	Formula Weight	Exact Mass	Molecular Formula	Yield (%)	Log P		
					Biovia	VCClab	ACD Chemsketh
MTX	4454.44	454.439	C_20_H_22_N_8_O_5_	-	0.11	−0.91	0.023 ± 0.83
DSPE-MTX	1184.49	1183.73	C_61_H_102_N_9_O_12_P	81.1	13.84	7.53	16.63 ± 1.03
PEG-MTX	1480.69	1479.80	C_67_H_11_7N_9_O_27_	78.3	−0.67	0.42	−0.43 ± 0.93

**Table 2:** Sizes, PdI, ζ-Potential and encapsulation efficiency (%EE) of the formulation used.

Liposomes	Size (nm)	Pdl	Z Pot (mV)	%EE
DSPE-MTX	159 ± 3	0.14 ± 0.02	−38 ± 0.26	79.9 ± 5.6
PEG-MTX	166 ± 0.6	0.18 ± 0.02	−41 ± 0.4	82 ± 7.5
Combo	148 ± 1	0.17 ± 0.01	−41 ± 3	80.2 ± 1.8
Empty	157.8 ± 2	0.17 ± 0.01	−41.84 ± 1.2	-

**Table 3:** R2 values of the zero-order, first-order kinetic, Higuchi models, Korsmeyer–Peppas and Weibull models. Kinetic parameters for the Korsmeyer–Peppas model: K represents the release rate constant; n represents the release mechanism of drug. Kinetic parameters for the Weibull model: a represents a constant based on the system, and b a constant based on the release kinetics.

Prodrug	Zero-Order	First-Order	Higuchi	Korsmeyer-Peppas	Weibull	K	n	a	b
DSPE-MTX	0.9796	0.9824	0.9004	0.9830	0.8905	6.80	0.9931	0.0527	1.3510
PEG-MTX	0.9648	0.9847	0.9821	0.9931	0.9672	13.85	0.64	0.1353	0.8829
DSPE-MTX_ PEG-MTX	0.9865	0.9942	0.9638	0.9966	0.9943	13.9	0.7506	0.1350	1.108

**Table 4:** IC_50_ of MTX, DSPE-MTX, DSPE-MTX-LIP, empty LIP, PEG-MTX, PEG-MTX-LIP, Combo and Combo-LIP calculated on BMDMs 72 h after treatment.

Sample	IC_50_ 72 h
MTX (μM)	24.1 ± 0.14
DSPE-MTX (μM)	0.9 ± 0.1
DSPE-MTX- LIP (μM)	0.7 ± 0.12
PEG-MTX (μM)	2.5 ± 0.08
PEG-MTX-LIP (μM)	1.6 ± 0.1
Combo (μM)	0.6 ± 0.1
Combo- LIP (μM)	0.9 ± 0.1
Empty LIP (μM)	-

## Data Availability

Data available within the article or its Supplementary Materials. Raw data are available on request from the authors.

## Supplementary Materials

The following are available online at https://www.mdpi.com/1999-4923/13/3/332/s1, Figure S1: 1H NMR spectrum of DSPE-MTX in CDCl_3_, Figure S2: 1H NMR spectrum of PEG-MTX in CDCl_3_, Figure S3: Transmission electron microscopy images of PEG-MTX-LIP, DSPE-MTX-LIP and Combo-LIP, respectively (scale bar: 100 nm), Figure S4: Mathematical models showing the fitting of DSPE-MTX-LIP. Results are representative of three independent experiments ± S.D. (*n* = 3), Figure S5: Mathematical models showing the fitting of PEG-MTX-LIP. Results are representative of three independent experiments ± S.D. (*n* = 3), Figure S6: Mathematical models showing the fitting of Combo-LIP. Results are representative of three independent experiments ± S.D. (*n* = 3), Figure S7: Physicochemical characterization of Cy5-LIP (A) and DSPE-CY5 release profile (B), Figure S8: Image reporting a maximum intensity profile of a z-stack of BMDMs treated with liposome reporting in blue the nuclei, in green the plasma membrane and in red the liposomes, split channel visualization.
