# Supplementary material for: Synthesis of Two Methotrexate Prodrugs for Optimizing Drug Loading into Liposomes
Source: Pharmaceutics. 2021 Mar 4;13(3):332. doi: 10.3390/pharmaceutics13030332 (PMC7998143; doi:10.3390/pharmaceutics13030332)
Supplement: Supplementary file 1 [file pharmaceutics-13-00332-s001.pdf]

pharmaceutics

Supplementary Materials: Synthesis of Two Methotrexate Prodrugs for Optimizing Drug Loading into Liposomes

Valentina Di Francesco, Martina Di Francesco, Paolo Decuzzi, Roberto Palomba and Miguel Ferreira

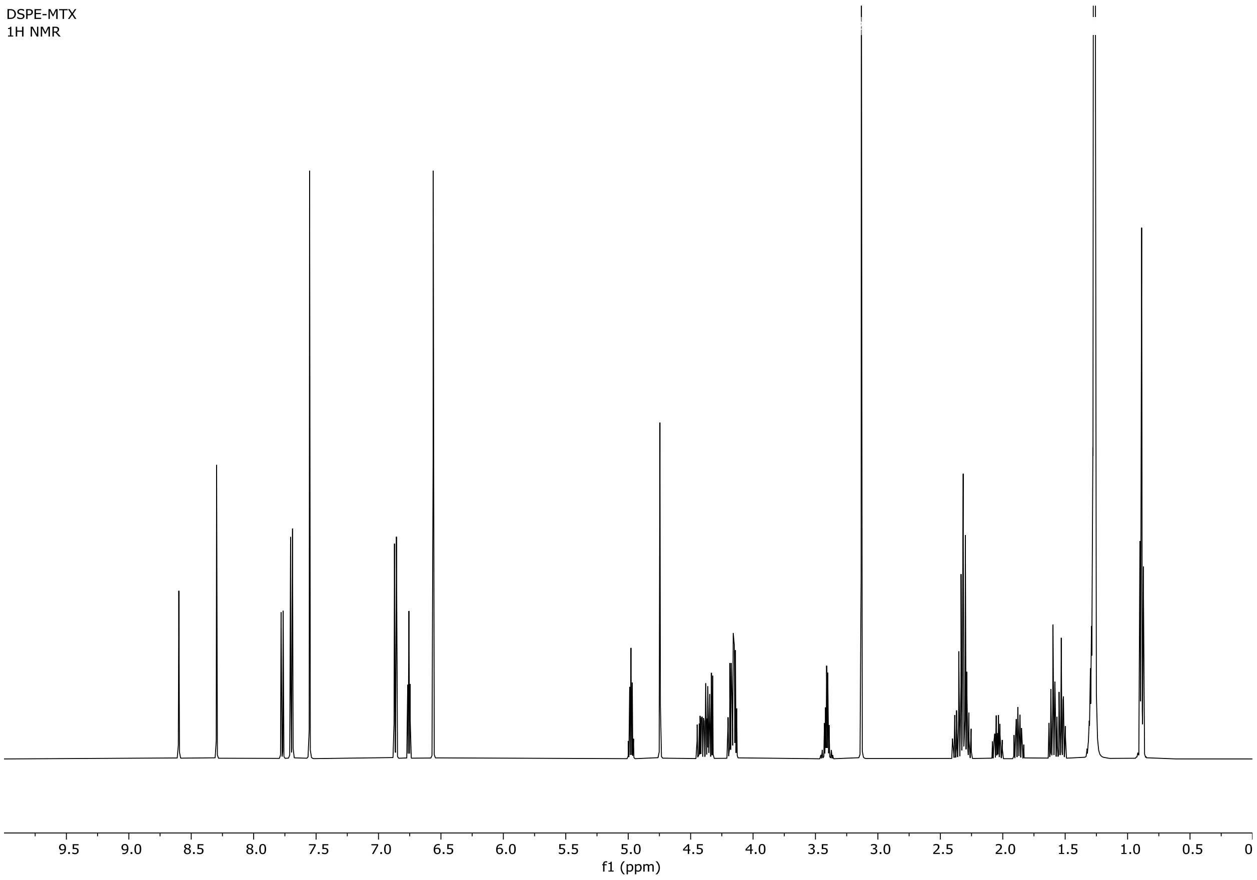

Figure S1. ^{1}H NMR spectrum of DSPE-MTX in $CDCl_3$.

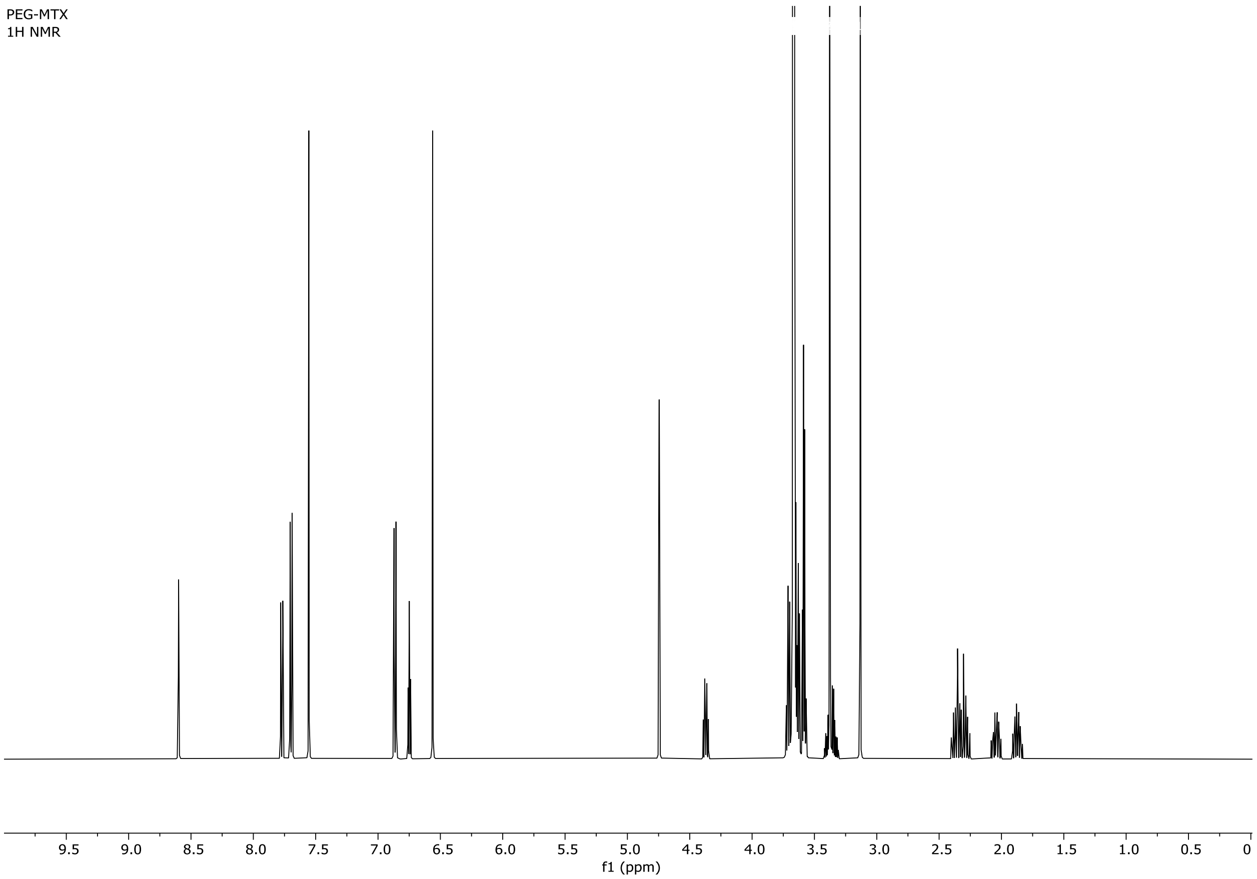

Figure S2. ^1H NMR spectrum of PEG-MTX in $CDCl_3$.

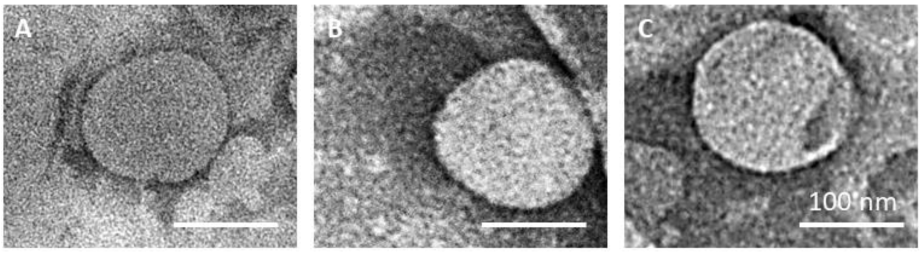

Figure S3. Transmission electron microscopy images of PEG-MTX-LIP, DSPE-MTX-LIP and Combo-LIP, respectively (scale bar: 100 nm).

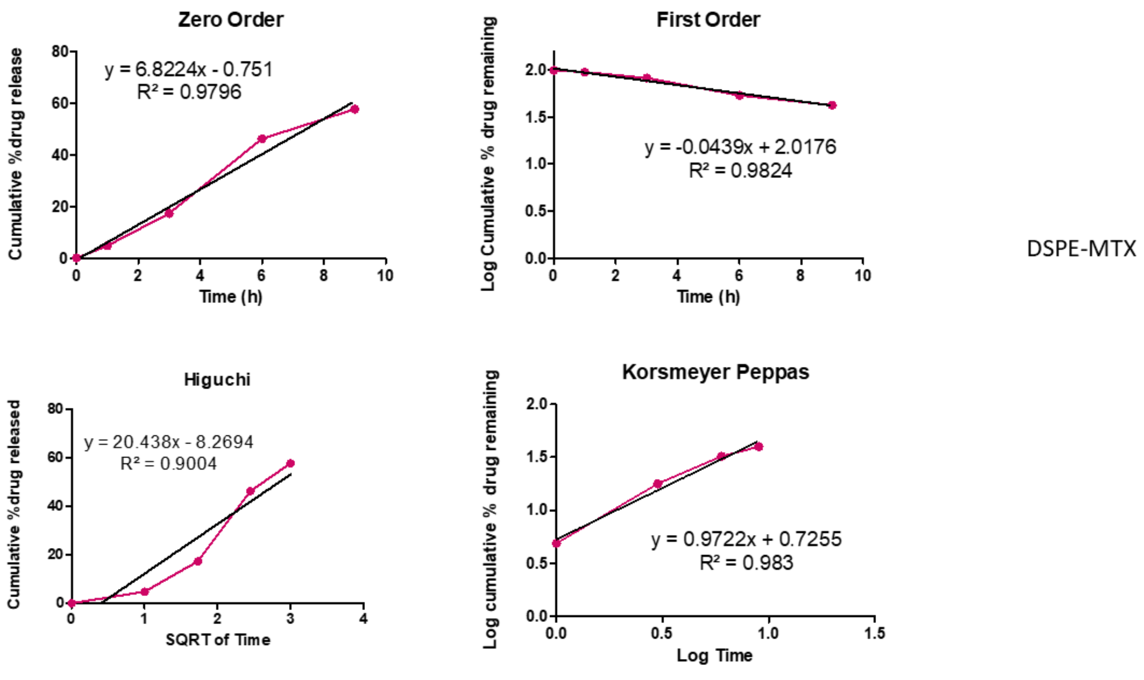

Figure 4. Mathematical models showing the fitting of DSPE-MTX-LIP. Results are representative of three independent experiments ± S.D. (n = 3).

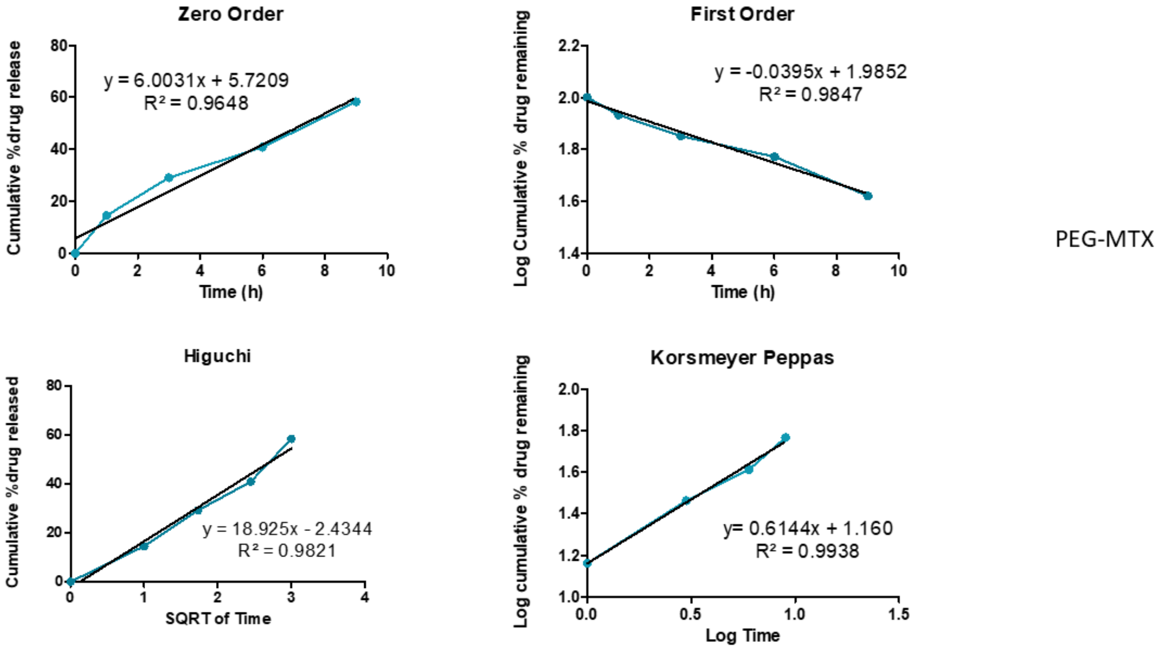

Figure S5. Mathematical models showing the fitting of PEG-MTX-LIP. Results are representative of three independent experiments ± S.D. (n = 3).

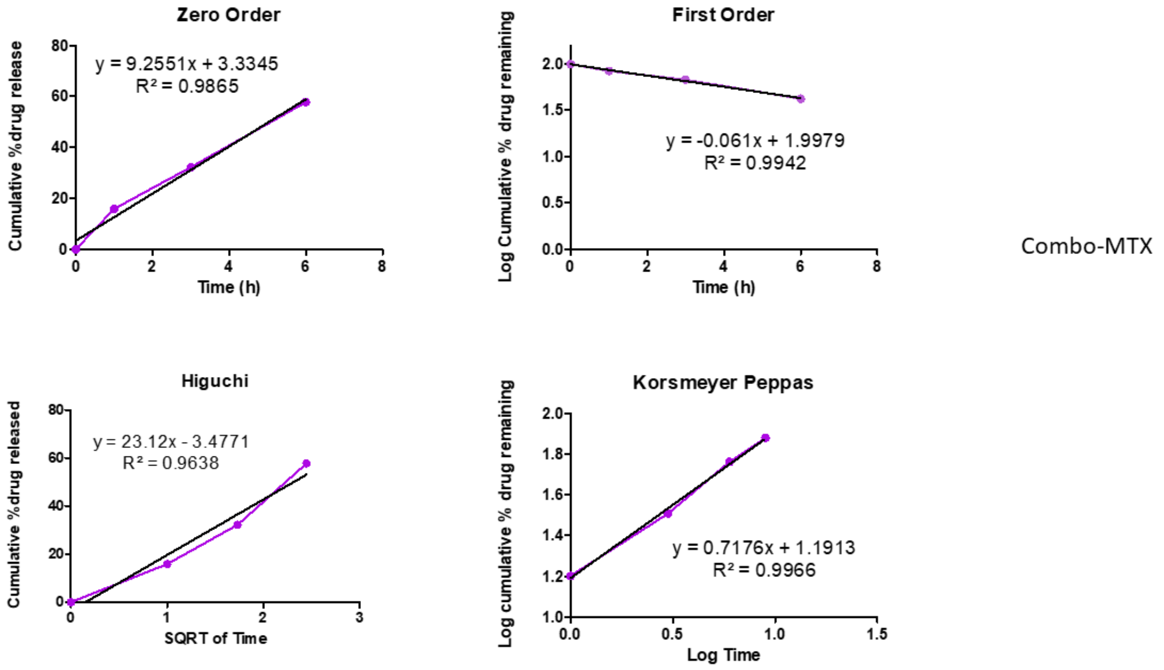

Figure S6. Mathematical models showing the fitting of Combo-LIP. Results are representative of three independent experiments ± S.D. (n = 3).

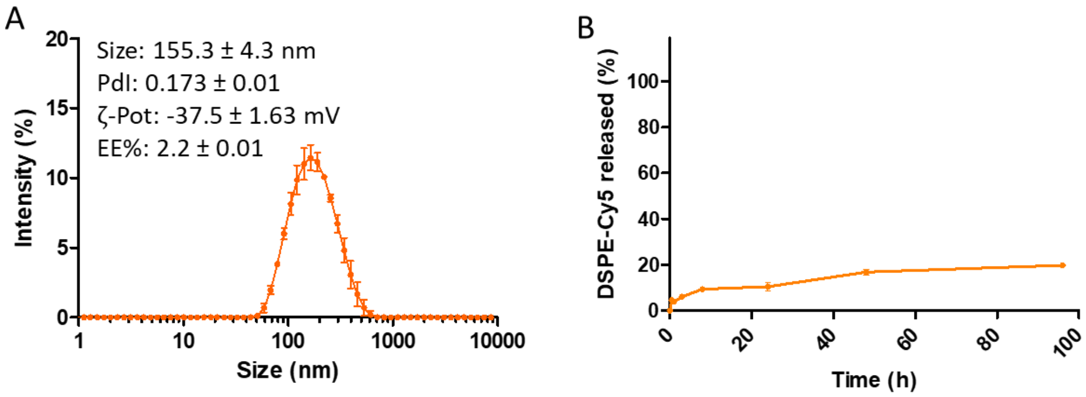

Figure S7. Physico-chemical characterization of Cy5-LIP (**A**) and DSPE-CY5 release profile (**B**).

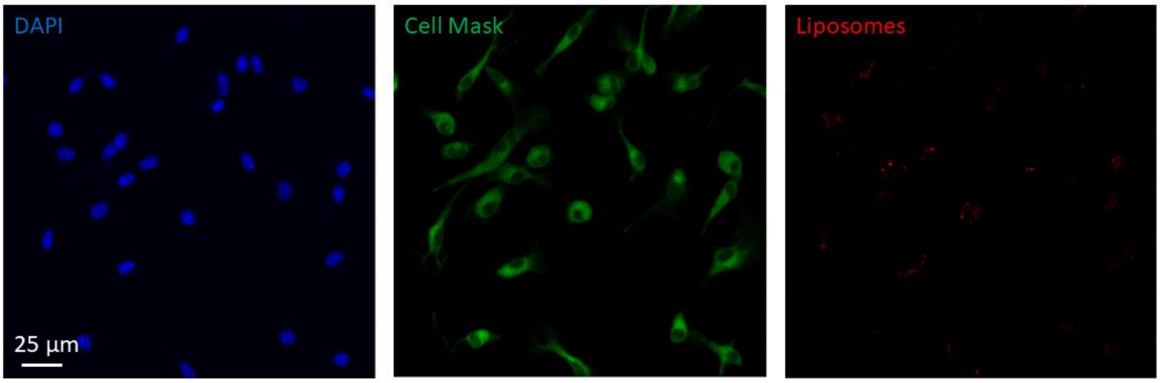

Figure S8. Image reporting a maximum intensity profile of a z-stack of BMDM treated with liposome reporting in blue the nuclei, in green the plasma membrane and in red the liposomes, split channel visualization.
